# Ultrasound-guided thoracostomy site identification in healthy volunteers

**DOI:** 10.1186/s13089-018-0108-1

**Published:** 2018-10-15

**Authors:** Lindsay A. Taylor, Michael J. Vitto, Michael Joyce, Jordan Tozer, David P. Evans

**Affiliations:** Virginia Commonwealth University Emergency Medicine, 1250 E Marshall Street, 2nd Floor, Suite 500, P.O. Box 980401, Richmond, VA 23298 USA

## Abstract

**Background:**

Traditional landmark thoracostomy technique has a known complication rate up to 30%. The goal of this study is to determine whether novice providers could more accurately identify the appropriate intercostal site for thoracostomy by ultrasound guidance.

**Methods:**

33 emergency medicine residents and medical students volunteered to participate in this study during routine thoracostomy tube education. A healthy volunteer was used as the standardized patient for this study. An experienced physician sonographer used ultrasound to locate a site at mid-axillary line between ribs 4 and 5 and marked the site with invisible ink that can only be revealed with a commercially available UV LED light. Participants were asked to identify the thoracostomy site by placing an opaque marker where they would make their incision. The distance from the correct insertion site was measured in rib spaces. The participants were then given a brief hands-on training session using ultrasound to identify the diaphragm and count rib spaces. The participants were then asked to use ultrasound to identify the proper thoracostomy site and mark it with an opaque marker. The distance from the proper insertion site was measured and recorded in rib spaces.

**Results:**

The participants correctly identified the pre-determined intercostal space using palpation 48% (16/33) of the time, versus the ultrasound group who identified the proper intercostal space 91% (30/33) of the time. On average, the traditional technique was placed 0.88 rib spaces away (95 CI 0.43–1.03), while the ultrasound-guided technique was placed 0.09 rib spaces away (95 CI 0.0–0.19) [P = 0.003].

**Conclusions:**

The ability to accurately locate the correct intercostal space for thoracostomy incision was improved under ultrasound guidance. Further studies are warranted to determine if this ultrasound-guided technique will decrease complications with chest tube insertion and improve patient outcomes.

## Background

The leading cause of death for individuals in the United States younger than 40 years old is trauma, with approximately 140,000 deaths annually [1]. Of these deaths, thoracic trauma accounts for nearly a quarter of all traumatic deaths [2]. Despite its severity, less than 30% of penetrating chest trauma and 10% of blunt thoracic injuries require thoracostomy [3]. In these patients, thoracostomy tube placement is performed to manage conditions such as pneumothorax, hemothorax, and pleural effusions. The goal of thoracostomy drainage remains unchanged since the time of Hippocrates; however, the procedure itself has changed dramatically since fifth century B.C.E [4]. In 1876, Hewett was the first to use a completely closed intercostal drainage system, [5] but it was not until World War II that tube thoracostomy became common in the treatment of injured patients [6]. From this point, thoracostomy tube placement became a mandatory skill for all providers taking care of trauma patients. Unfortunately, this life-saving procedure has a complication rate up to 30% [7], with complications raging from tube malposition, to bleeding, and organ injury [8–11].

To prevent these risks, training programs design simulations to train their residents on the proper insertion techniques. The classic technique uses landmarks on the body to identify the anatomically correct insertion site to avoid intraperitoneal insertion or lung injury. One study evaluated 50 junior physicians’ ability to properly identify the chest thoracostomy site using a photograph of a chest wall and showed that only 44% correctly located the 4th–5th intercostal space in the mid-axillary line [12]. Another study had physicians placing a radiopaque marker on a patient’s chest wall over the 4th or 5th ICS prior to the patient getting a chest X-ray. This study demonstrated that physicians placed the marker correctly only 36.2% of the time [13]. In both of the aforementioned studies, the most common mistake made by the trainees was placing the marker for thoracostomy insertion too low [12, 13].

In many institutions, thoracic ultrasound is required prior to intervention for suspected pleural fluid, whether simple pleural effusion, hemothorax or other etiology to reduce the risk of iatrogenic complications during pleural interventions [14].

We designed a pilot study to test if ultrasound guidance improved proper thoracostomy site identification over traditional landmark technique in a healthy volunteer.

## Methods

### Study design

This was a simulation-based study in which observational results were collected during routine thoracostomy tube training of novice providers. The Institutional Review Board approved this study protocol as exempt.

### Study population

33 emergency medicine residents volunteered to take part in this study. The participants had varied levels of skill ranging from no experience with thoracostomy placement to senior residents with greater than 10 successful thoracostomy placements. All residents participate in a 16-h ultrasound course at the beginning of their intern (training) year. This course covers echocardiography, thoracic ultrasound, and trauma (eFAST) along with the American College of Emergency Physicians (ACEP) core topics. Following ACEP guidelines, and to ensure the residents are competent in ultrasound, they must perform good-quality scans in several categories upon completion of their training. The categories pertinent to this study include: 25 eFAST, 25 thoracic, and 50 Echocardiograms.

The participants were instructed and evaluated by dedicated clinical ultrasound faculty and received no direct incentive for participating in the study.

### Study protocol

A healthy volunteer was used as the standardized patient for this study. The correct thoracostomy site was defined as the mid-axillary line between ribs 4 and 5. The correct site was pre-identified on both sides by two experienced physicians and marked with invisible ink that could only be revealed with a UV LED flashlight.

The study was performed in two stages. The first stage of the study evaluated the participant on the placement of the chest tube using traditional landmarks. After locating the site, the participant was instructed to place a plastic arrow over the chest tube insertion site. The plastic marker was compared to the pre-identified mark using the UV LED light, and if the plastic marker was incorrectly positioned, the difference in ribs spaces was recorded.

**Fig. 1:**
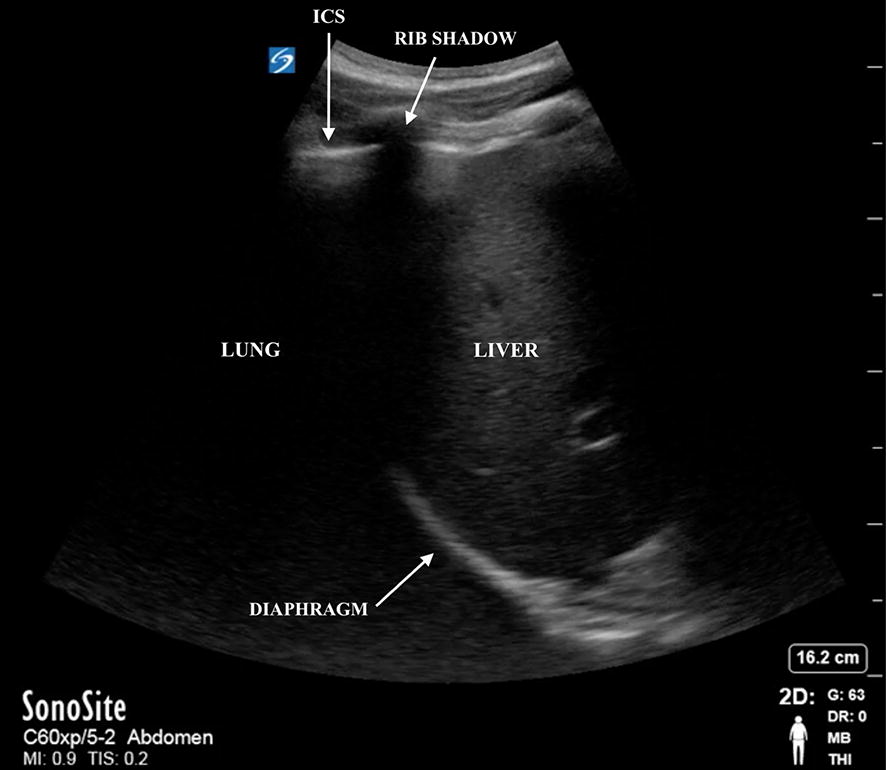
This is a right upper quadrant ultrasound image at the costophrenic angle demonstrating the starting point of the ultrasound-guided identification of the diaphragm, ribs, and intercostal space

**Fig. 2:**
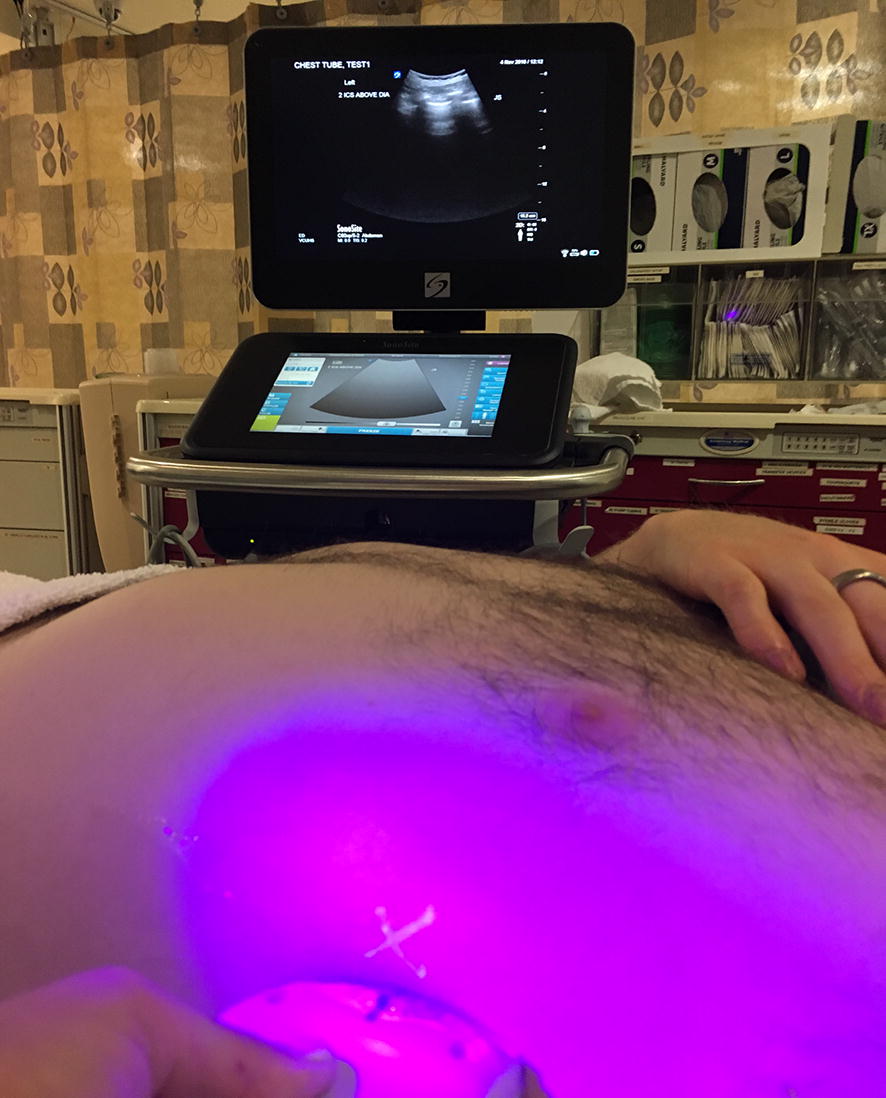
The curvilinear transducer placement in the correct intercostal space. The LED flashlight has illuminated the landmark (represented by the “X” on the volunteer’s skin). On the ultrasound screen in the background, the ICS is demonstrated in the center of the screen between two rib shadows

The second stage of the study involved a simulation-based approach to teach the participants how to identify the diaphragm as well as identify and count adjacent rib spaces using ultrasound. The participants were given a brief hands-on teaching session using a curvilinear transducer with the indicator pointing cephalad. The transducer was placed at the costophrenic angle on the mid-axillary line as a starting point. Once the diaphragm was identified, the participant was instructed to look for diaphragmatic excursion and track the diaphragm to the near field on the screen where rib shadows would be present (Fig. 1). The ICS adjacent to the first rib cephalad to the diaphragm was designated as ICS zero. Sliding the transducer superiorly 2 additional ribs spaces accurately located the ICS between the 4th and 5th ribs. Once the participant was taught the technique, they were tested. The participant was asked to use the curvilinear transducer to identify the correct ICS and hold the middle of the transducer over the insertion site. The insertion site was then compared to the pre-identified site by a UV LED light (Fig. 2).

### Equipment

A SonoSite X-Porte ultrasound machine with a curvilinear C60XP 5-2 MHz transducer (SonoSite, Inc, Bothell, WA). TaoTronics 12 UV LED flashlight, model TT-FL001. UV LED flashlight. Reactive invisible ink, nontoxic, fine point pen. Healthy volunteer standardized patient with a BMI of 24.4.

### Data collection

The faculty instructors were responsible for recording the data during the study. The number of rib spaces that deviated from the correct ICS was recorded and the data were entered into an Excel spreadsheet (Microsoft Corporation, Redmond, WA). The results were analyzed using a single -ailed student *t* test. After the completion of the study, the participants were asked to fill out a short survey regarding perceived effectiveness of study and its applicability to identify the ideal ICS for thoracostomy placement. The survey choices given to the participant ranged from strongly disagree, disagree, neutral, agree, to strongly agree.

## Results

**Fig. 3:**
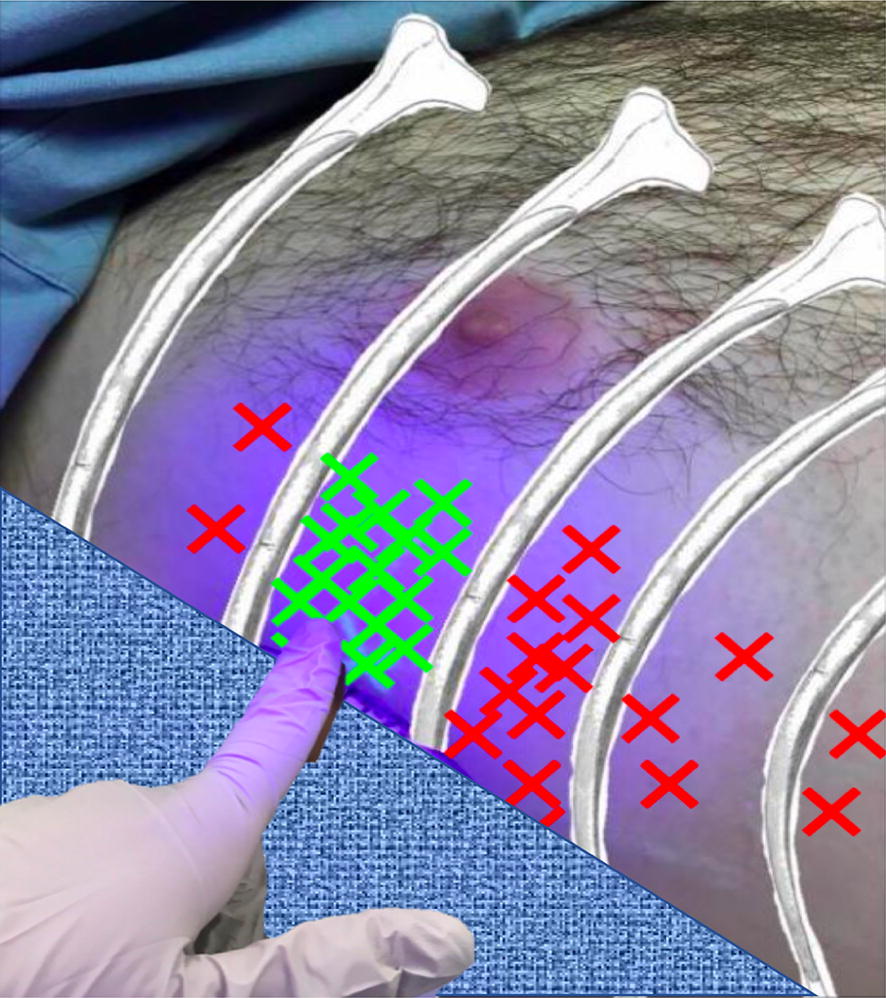
This is an illustrative representation of the data plotted on the volunteer’s chest wall for the landmark-palpated method. The green “X’s” represent the correct intercostal space, whereas the red “X’s” demonstrate the number of participants who did not place the marker in the correct ICS. The data plotted were illustrated and represented in rib spaces above and below the target

In the first stage of the study, the landmark-palpated site was accurate at the level of the pre-determined mark 48% (16 of 33) of the time [95 CI 0.43–1.03]. Of these 17 improperly located marks, 12 were 1 ICS off, 3 were 2 ICS off, and 2 were 3 ICS off (Graph 1, Fig. 3). The standard deviation using the traditional technique was 0.88 rib spaces away.

**Fig. 4:**
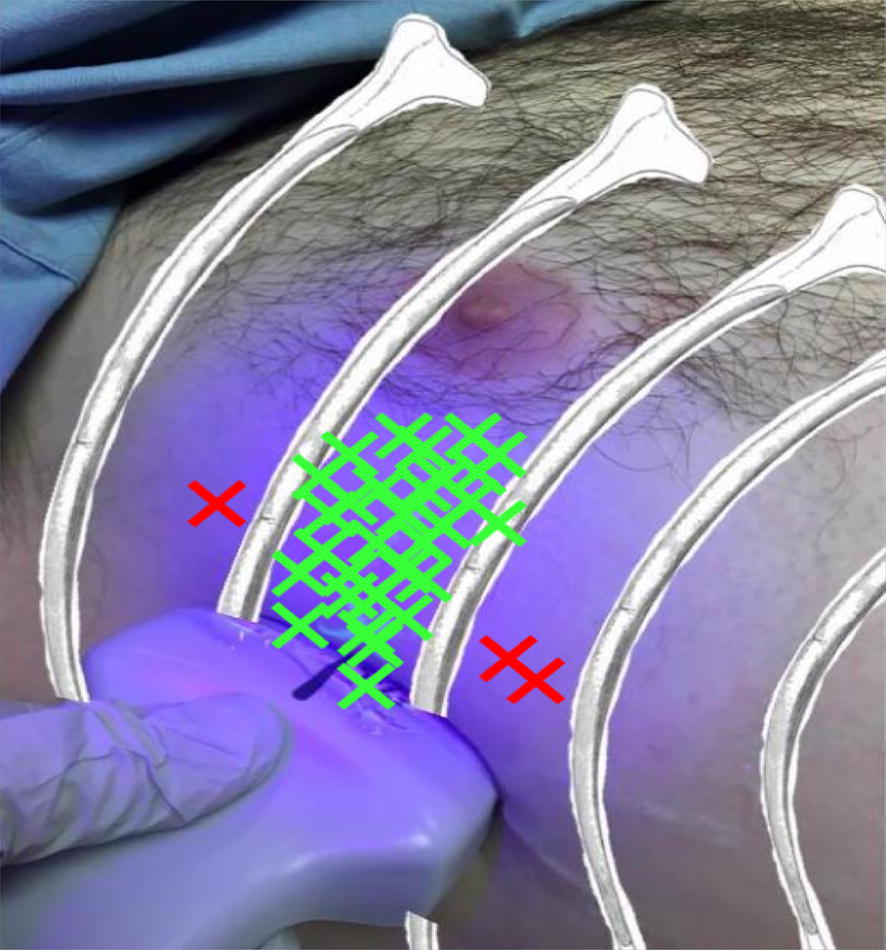
This is an illustrative representation of the data plotted on the volunteer’s chest wall for the ultrasound-guided method. The green “X’s” represent the correct intercostal space, whereas the red “X’s” demonstrate the number of participants who did not place the transducer in the correct ICS. The data plotted were illustrated and represented in rib spaces above and below the target ICS

The second stage of the study tested the accuracy of the same 33 participants after a brief hands-on session on identifying the correct ICS under US guidance. The participants correctly identified the pre-determined mark 91% (30 of 33) of the time using ultrasound. Of the 3 improperly located marks, all 3 were 1 ICS off (Graph 1, Fig. 4). The standard deviation using the ultrasound-guided technique was 0.09 rib spaces away (95 CI 0.0–0.19) [p = 0.003].

## Discussion

Chest thoracostomy is a lifesaving procedure in patients with thoracic trauma. Unfortunately, this procedure is not without risks and is associated with significant morbidity and even mortality related to complications of its placement [15–17].

Ultrasonography is a well-established modality with the ability to reduce errors and increase success rate for many emergency medicine procedures [18, 19]. During this study, ultrasound was used to guide placement of the thoracostomy tube and serve as an adjunct to traditional methods in helping to identify the correct ICS, location of the diaphragm and any underlying solid organs.

While other studies challenged learners to place thoracostomy tubes within the “triangle of safety,” this study challenged the participants to be more precise and locate the 4th or 5th intercostal space. [12, 20] Of note, the simulated model had normal anatomy with no anatomic variation to the level of his diaphragm; therefore, localizing the precise intercostal space was part of this protocol. This exact methodology may not always apply in real-life situations, as patients may have a variation in the level of the diaphragm secondary to medical conditions such as COPD, phrenic nerve palsy, or increased intraabdominal pressure (e.g., pregnancy or ascites). With this consideration, simply identifying the diaphragm and moving 2–3 intercostal spaces cranially will localize a safe site for tube thoracostomy placement.

Ultrasound showed a significant improvement in the ability to identify the correct thoracostomy site. The traditional landmark technique identified the correct thoracostomy site only 48% of the time versus 91% in the ultrasound-guided group.

A post study anonymous survey demonstrated that all participants “agreed” or “strongly agreed” that ultrasound-guided thoracostomy site identification was easy to learn and useful in improving chest thoracostomy site identification. They also “agreed” or “strongly agreed” that they will consider incorporating this adjunct in all patients who require chest thoracostomy, particularly patients with difficult landmarks or a concern for a displaced diaphragm.

For the novice provider, ultrasound can increase the ability to identify the correct intercostal space compared to traditional landmark-guided techniques. Given the expanding use of ultrasound in clinical practice (diagnostic and interventional), this technique may serve as a useful adjunct to the identification of a safe zone for chest thoracostomy. In turn, this interventional ultrasound approach could be added to the ultrasound curricula when teaching critical care ultrasound.

### Limitations

This study has several limitations. The simulated nature of the study carried out in a controlled setting is inherently different than the clinical environment in which thoracostomy tubes are placed. Our standardized patient had a normal BMI with palpable landmarks and a normal anatomic location of his diaphragm. It is known that thoracostomy complication rate increases in the obese patient [21]. Despite these limitations, it appears ultrasound could still serve as a useful adjunct to thoracostomy site identification. Another limitation in this study is the lack of subsequent evaluation of the residents to verify if they retained the skills of this educational intervention. Despite the absence of re-evaluation, the residents train at an urban trauma center and perform several eFAST examinations on a clinical shift. This reinforces the skill of identifying the right and left thoracoabdominal interface with each study.

### Conclusion

In this pilot study, the ability of novice providers to accurately locate the correct intercostal space for chest tube insertion was improved under ultrasound guidance. The data from this study add to the growing body of evidence that physicians have a high rate of misplaced thoracostomy insertions. This novel technique can greatly improve proper thoracostomy site identification in the clinical setting.
